# Female Attendings in University Clinics of Surgery in Germany: A Scoping Analysis of an Ongoing Disparity

**DOI:** 10.7759/cureus.60860

**Published:** 2024-05-22

**Authors:** Mona Bickel-Dabadghao, Yannick Rau, Ludwig Matrisch

**Affiliations:** 1 Medicine, University of Lübeck, Lübeck, DEU; 2 General Practice, General Practice Teetzmann, Mölln, DEU; 3 Internal Medicine, University Hospital Schleswig-Holstein, Campus Lübeck, Lübeck, DEU

**Keywords:** healthcare system, discrimination, gender, germany, education, training, gender disparities, surgery

## Abstract

Introduction: Gender-based discrimination, particularly in healthcare, affects women’s roles and opportunities, including in surgery where they remain underrepresented in leadership positions. The extent to which such discrimination is prevalent in attending positions is unclear.

Methods: The publicly available records of 48 universities and university-associated hospitals in Germany were extracted to quantify gender ratios among attending surgeons and head surgeons in the fields of visceral, vascular, cardiac, thoracic, pediatric, orofacial, neuro-, trauma, and plastic surgery. Statistical analysis, including Chi-Square tests and Student’s t-test, was used to analyze the data.

Results: Among the 367 department heads, 353 (96.2%) were male and 14 (3.8%) were female. Among the 2,366 attendings, 1,854 (78.4%) were men and 512 (21.6%) were women. These differences were significant (χ²=64.95, p<0.001, odds ratio=0.14, 95% confidence interval=0.08-0.25). Departments being led by a female department head were not more likely to employ female attendings (χ²=0.379, p=0.538, odds ratio=1.17, 95% confidence interval=0.70-1.96).

Conclusion: German surgical departments in University Hospitals have significant gender disparities, with women underrepresented at higher levels. This may negatively affect patient outcomes. To tackle the problem, further research is needed to fully understand the issue.

## Introduction

Discrimination of women is a multifaceted and ongoing issue in societies around the world with a centuries-old legacy. In an international context, women are more affected by limited access to education as well as lower social status and less economic independence in comparison to men [1]. Healthcare as a field is particularly permeated by gender norms regarding not only the physician’s role but also non-physician healthcare staff. Thus, it is hardly surprising that gender-based prejudice also impacts physician top-level employment [2]. Although women have been active in surgery for over 5,500 years, to this day, they still do not hold an equal role as men [3].

Any organization is substantially shaped by the workers in its leading positions [4]. Therefore, to analyze discrimination within an organization, a quantitative analysis of its leaders is important. However, such analysis of surgical departments in German university hospitals has not been conducted yet.

Surgeons in positions of additional authority, who are also working in a managing capacity, are of special interest in this regard. In Germany, these surgeons in a leading and managing capacity are called “Oberarzt” (male) or “Oberärztin” (female) which can be roughly translated to attending or consultant surgeons in English.

In this study, we aim to determine how gender-based discrimination shaped surgical departments by analyzing gender disparities at the attending level. We provide potential explanations for these disparities and point toward solutions to tackle the problem.

## Materials and methods

Data collection

Data were collected in November 2022. All attendings (Oberärzt*innen) and department heads working in surgical departments in German university hospitals were included. We extracted the data from the publicly available websites of the hospitals. The surgical specialty was stated on the website in most cases. Secondary online sources were used to determine the specialty if it was not stated on the website. Surgeons were grouped according to all purely surgical specialties available for residency in Germany [5]. Only specialties associated with the German Society of Surgery were included, resulting in the exclusion of specialties traditionally considered surgical, i.e., urology, and obstetrics and gynecology. Gender was either stated on the website or determined by name. Hospitals not listing their employed surgeons on their websites were asked to share these data in a formal inquiry.

The data from 48 university and university-associated hospitals in Germany were gathered to quantify the number and gender of attending surgeons and department heads in the fields of visceral, vascular, heart, thoracic, pediatric, orofacial, orthopedic and trauma, neuro- and plastic surgery. University hospitals were classified as such if they held membership in the Association of German University Hospitals [6].

Not all university hospitals or associated hospitals employ all included departments and were, in their respect, only included in the analysis of each department that they were facilitating. Only administratively separated departments were included in the analysis. Subdivisions within a department were considered to be part of the respective department and not analyzed separately. Departments that had more than one head surgeon appointed were also included and each head surgeon was considered to be equal in position to each other and to those in other hospitals’ departments.

Statistical analysis

Statistical analysis was performed using the R-based Software Solution Jamovi (Version 2.2.5, The Jamovi Project, Sydney, Australia). To assess group differences in mean male-to-female ratios, a Student’s t-test was performed. To analyze the dependence between variables like the gender ratio and department, Chi-square test was performed. For all analyses, an alpha error probability lower than p<0.05 was considered to be significant.

## Results

**Figure 1:**
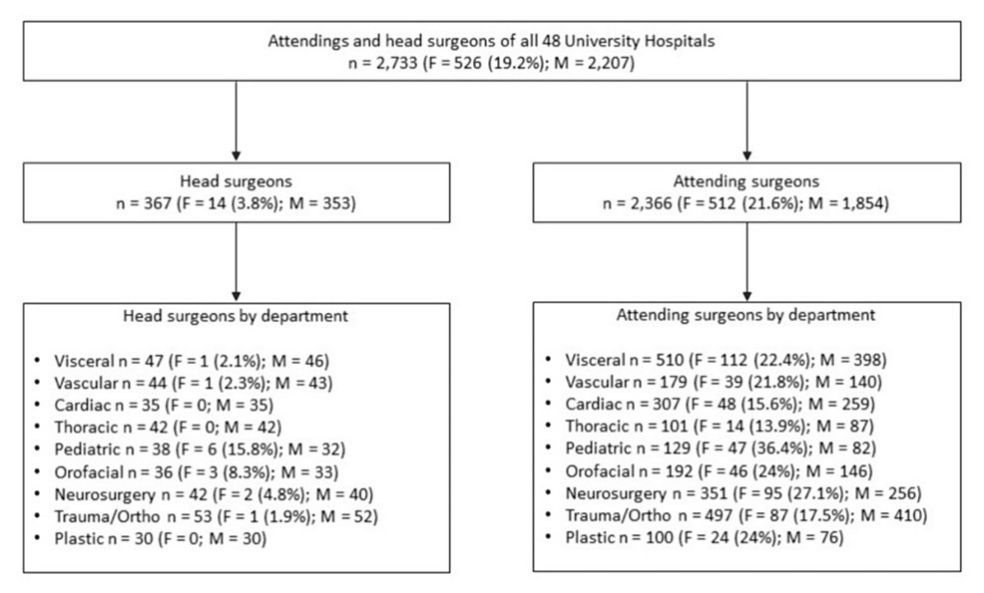
University head and attending surgeons by department Abbreviations: M=Male, F=Female

Forty-eight university hospitals were included in this study. Those employed 2733 surgeons either as head surgeons or as attendings. Within the 367 department heads, 353 (96.2%) are male, while 14 (3.8%) are female. While none of the plastic surgery and cardiac surgery departments are led by a woman, six out of 38 (15.8%) pediatric surgery departments are under female leadership. One thousand eight hundred fifty-four out of 2,366 attendings (78.4%) are men, while 512 (21.6%) are women. In thoracic surgery, 14 out of 101 (13.9%) attendings are female, whereas 47 out of 129 (36.4%) attendings in pediatric surgery are women (Figure 1). 

**Figure 2:**
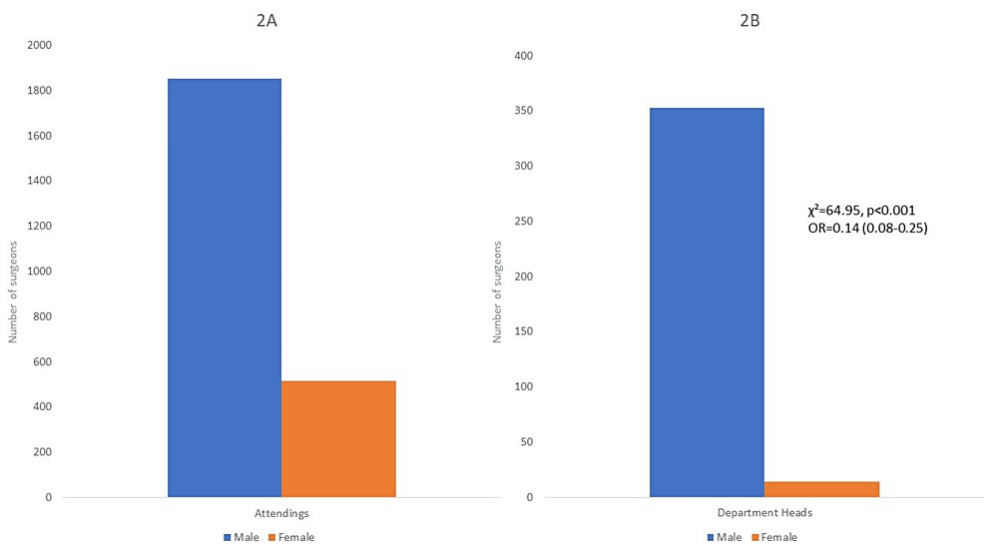
Number of female and male surgeons in attending positions (A) and department head positions (B) The results of a Chi-Square test using the gender and the position as variables are provided on the right side. The numbers in brackets signify the 95% confidence interval for the odds ratio. Abbreviations: OR=Odds ratio

To assess the permeability to leading positions in the surgical departments, we conducted a Chi-square test using the gender as well as the position of the surgeons as variables. χ² was 64.95 with p<0.001. The odds ratio was 0.14 (95% Confidence Interval=0.08-0.25) (Figure 2).

We assessed whether a department being led by a female head was associated with a higher proportion of female attendings working in that department using a Chi-square test. The findings were insignificant (χ²=0.379, p=0.538) with an odds ratio of 1.17 (95% confidence interval=0.703-1.96).

## Discussion

To the best of our knowledge, this is the first study to assess gender disparities in surgical departments of German university hospitals stratified by specialty. The data provided in this article reveal gender disparities at the attending level and more so at the level of the department heads. The gap between the attending and the department head level implies a lack of permeability, the so-called “glass ceiling,” for women.

To understand the data’s implications, one needs to grasp the concept behind the German system of medical education in medical school as well as in residency and during the fellowship. Medical students can start medical school right after receiving their high school diploma, which is usually at the age of 17 to 19 years old.

Medical school lasts six years and includes mandatory rotations in surgery, including but not limited to a mandatory four-month rotation during the last year of study. Additionally, students can choose surgical specialties during their elective rotations (so-called “Famulatur”). Residency programs in surgery last five to six years [5]. Doctors can choose between specializations in general surgery, vascular surgery, cardiac surgery, pediatric surgery, orthopedics and traumatology, plastic surgery, thoracic surgery, oro-maxillofacial surgery, or visceral surgery. Specifics about residency vary slightly between states. The information given here applies to the state of Schleswig-Holstein since the authors are based in that state. The residency is completed with an oral exam and leads to board certification in the specific field chosen. Board certification qualifies but does not entitle doctors to attending positions.

Grasreiner et al. repeated cross-sectional surveys on medical students at the University of Jena, Germany, to determine their specialty preferences during the study [7]. During all three years examined, women showed a higher preference to work in a surgical field after their studies in comparison to men. In 2014, 22.2% of women and 19.3% of men wanted to specialize in surgery, those numbers were 28.3% for women and 24.1% for men in 2015, and even 30.4% for women and 15.2% for men in 2016. It is noteworthy that, in this study, gynecology and obstetrics, and urology fall under the umbrella of surgical specialties, the former of which is notorious for being a “female specialty” [8]. However, even excluding obstetrics and gynecology, 23.6% of female medical students in 2016 were interested in pursuing a surgical career path. Although the authors do not consider these gender disparities to be significant due to the small sample size, one can conclude that the lower number of female attendings is unlikely to be a result of less interest in surgical specialties by female medical students. This holds especially true considering that throughout Germany, around two-thirds of medical students are female [7,9,10].

The normative argument against discrimination based on gender has been made many times and shall not be the focus of this article [11,12]. We rather concentrate on the adverse outcomes this discrimination has on healthcare for patients. Wallis et al. found that in 1,320,108 analyzed cases, sex discordance was a predictor for worse outcomes in common surgical procedures [13]. Female patients treated by male surgeons were 15% more likely to suffer from adverse postoperative outcomes in comparison to female patients treated by female surgeons. These effects were the strongest in cardiac surgery as well as vascular surgery [13]. In German universities and university-affiliated hospitals, these two fields have three times more males than female attendings. Since attendings usually are the lead surgeons, especially in more complex cases, we infer that the number of surgeries performed on female patients with surgeon-patient sex discordance is higher than it was with more female attendings. Thus, gender inequality at the attending level may lead to worse patient outcomes. Also, female surgeons’ style of communication and counseling tends to be perceived as better by patients [14]. This tends to build better relationships with patients and is associated with better therapy outcomes [15].

Reasons

The underlying reasons for the gender disparities described in this study are manifold. Among the reasons quoted by medical students for their choice of specialty are their academic interest in that specialty, their competencies, the lifestyle and work schedules associated with that specialty, patient service orientation, mentors, career opportunities, workload, income, length of training, prestige, and advice from others [16]. In the following sections, we will lay out how women are disproportionately affected in most of these areas in surgery and how that might influence their choice of a career in the field.

Surgery is considered one of the most demanding specialties in medicine. Surgeons are expected to work long hours while performing mentally and physically exhausting tasks [17]. This leads to a high prevalence of mental problems such as emotional exhaustion, depersonalization, and eventually burnout [18-20]. Such chores become more time-consuming when entering motherhood, often leaving women to decide between motherhood and career advancement.

Under the German maternity protection act, a surgeon’s work environment and structure are heavily affected by those protections, specifically regarding work time restrictions, exposure to embryo-, fetotoxic, and infectious substances, and restrictions on standing for long periods of time. Female surgeons have criticized that some of those restrictions are interpreted overly cautiously, e.g., banning pregnant women from performing surgery during the entirety of pregnancy, leading to reduced learning opportunities [21].

The long hours and high levels of commitment expected in surgery can be challenging for surgeons who are also parents. The propensity for part-time work is one factor that can influence the career paths of female surgeons. Balancing the demands of a surgical career with the responsibilities of parenthood often requires flexibility in work schedules. However, part-time work in surgery is not always feasible due to the nature of surgical procedures and patient care needs [22].

Childcare is another significant challenge for female surgeons. The demanding schedule of a surgeon can make it difficult to manage childcare responsibilities. This is particularly true for single parents or in situations where both parents have demanding careers [23]. Furthermore, the lack of adequate childcare facilities or support systems within the hospital or clinic can exacerbate these challenges [24].

Besides these factors, there are other systemic and cultural issues that can affect female surgeons. For instance, there may be unconscious bias or discrimination in the workplace, which can affect career progression and opportunities [25]. Additionally, societal expectations and norms around gender roles and parenting can influence the experiences of female surgeons.

Attendings in surgery play an important role in surgical departments, as they usually are specialists in one field and take responsibility for performing complex surgical procedures. Therefore, the amount and complexity of surgical procedures performed during training is an important factor influencing career development. However, female surgeons tend to have lower case volumes as well as less complex cases in comparison to their male counterparts [26]. These disparities persist after accounting for influencing factors such as seniority and specialty and cannot be explained by competing professional and familial obligations [27]. Thus, it seems likely that discrimination solely based on gender plays a role in these disparities.

These disparities have the potential to perpetuate because they lead to fewer women in leading roles, which could shape gender roles and thereby influence administrative decisions in surgical departments [28]. This lack of access to leading positions is often reflected by lower wages for women. This has been observed in various countries [29,30]. Specific data for the situation in Germany is scarce. It can, however, be assumed that the wage gap is smaller in comparison to other countries due to a high degree of commitment to collective bargaining in university clinics. Department heads, however, are exempt from collective salary agreements, so it is unclear how the wage gap affects male vs. female department heads.

 Limitations to our methods

Although we have acquired the data with the utmost diligence, there might be potential problems with our method. Since we used the clinics’ websites as our primary data source, we had to rely on the surgical departments to display their staff accurately. It might be possible this might not always be the case. If there were a systemic gender bias in the clinics’ presentation of their staff, this would influence our results. Also, we solely can state the physicians’ official position in their department. The actual power dynamics within the department that might play an important role cannot be assessed this way. The cross-sectional design of our study prevents us from making statements about current developments concerning gender equality in surgical departments of German University Hospitals. This includes assessing whether the gap between the attending and the department head level is the expression of a glass ceiling or other underlying reasons. Also, the effect of a woman leading the department on the development of gender disparities is still not clear. Further research with a more longitudinal study design is needed to assess whether the inequality currently aggravates or whether it diminishes.

## Conclusions

There currently are massive gender disparities within surgical departments of German university hospitals. Women are underrepresented at the attending and even more so at the department head level. This might be harmful to the treatment outcome of patients in those departments. We have laid out potential structural reasons for this situation. However, more research is needed to further understand the underlying mechanisms.
